# Supplementary material for: The effect of intrapartum prolonged oxygen exposure on fetal metabolic status: secondary analysis from a randomized controlled trial
Source: Front Endocrinol (Lausanne). 2023 Jun 27;14:1204956. doi: 10.3389/fendo.2023.1204956 (PMC10335765; doi:10.3389/fendo.2023.1204956)
Supplement: Supplementary file 3 [file DataSheet_3.zip › Result-X101SC21092977-Z01-J001-B1-42/4.MetDiffAnalysis/O.vs.A/O.vs.A_pos_zscore.pdf]

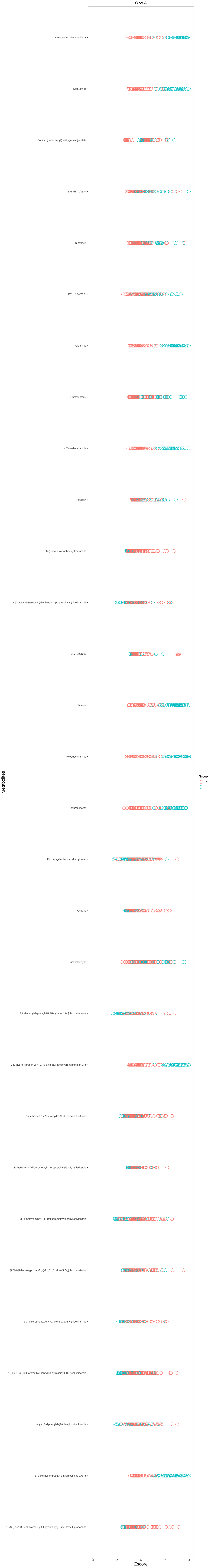

O.vs.A
trans,trans-2,4-Heptadienal
Stearamide
Sodium [dodecanoyl(methyl)amino]acetate
SM (d17:1/15:0)
Riboflavin
PC (18:1e/20:2)
Oleamide
Ohmefentanyl
N-Tetradecanamide
Nobiletin
N-(2-morpholinophenyl)-2-furamide
N-[2-acetyl-5-(tert-butyl)-3-thienyl]-2-(propylsulfanyl)nicotinamide
JNJ-1661010
Isophorone
Hexadecanamide
Fenpropimorph
Dihomo-γ-linolenic acid ethyl ester
Cytisine
Cuminaldehyde
8,8-dimethyl-2-phenyl-4H,8H-pyrano[2,3-h]chromen-4-one
7-(2-hydroxypropan-2-yl)-1,4a-dimethyl-decahydronaphthalen-1-ol
6-methoxy-2,3,4,9-tetrahydro-1H-beta-carbolin-1-one
3-phenyl-5-[3-(trifluoromethyl)-1H-pyrazol-1-yl]-1,2,4-thiadiazole
3-(dimethylamino)-2-[3-(trifluoromethyl)phenyl]acrylonitrile
(2S)-2-(2-hydroxypropan-2-yl)-2H,3H,7H-furo[3,2-g]chromen-7-one
2-(4-chlorophenoxy)-N-(2-oxo-3-azepanyl)nicotinamide
2-{(3S)-1-[4-(Trifluoromethyl)benzyl]-3-pyrrolidinyl}-1H-benzimidazole
1-allyl-4,5-diphenyl-2-(2-thienyl)-1H-imidazole
17α-Methyl-androstan-3-hydroxyimine-17β-ol
1-[(3S)-3-(1,3-Benzoxazol-2-yl)-1-pyrrolidinyl]-3-methoxy-1-propanone
Metabolites
-4
-2
0
2
4
Zscore
Group
A
O
